# Supplementary figures and images for: Prognostic Value of Necroptosis-Related Genes Signature in Oral Squamous Cell Carcinoma
Source: Cancers (Basel). 2023 Sep 13;15(18):4539. doi: 10.3390/cancers15184539 (PMC10527362; doi:10.3390/cancers15184539)

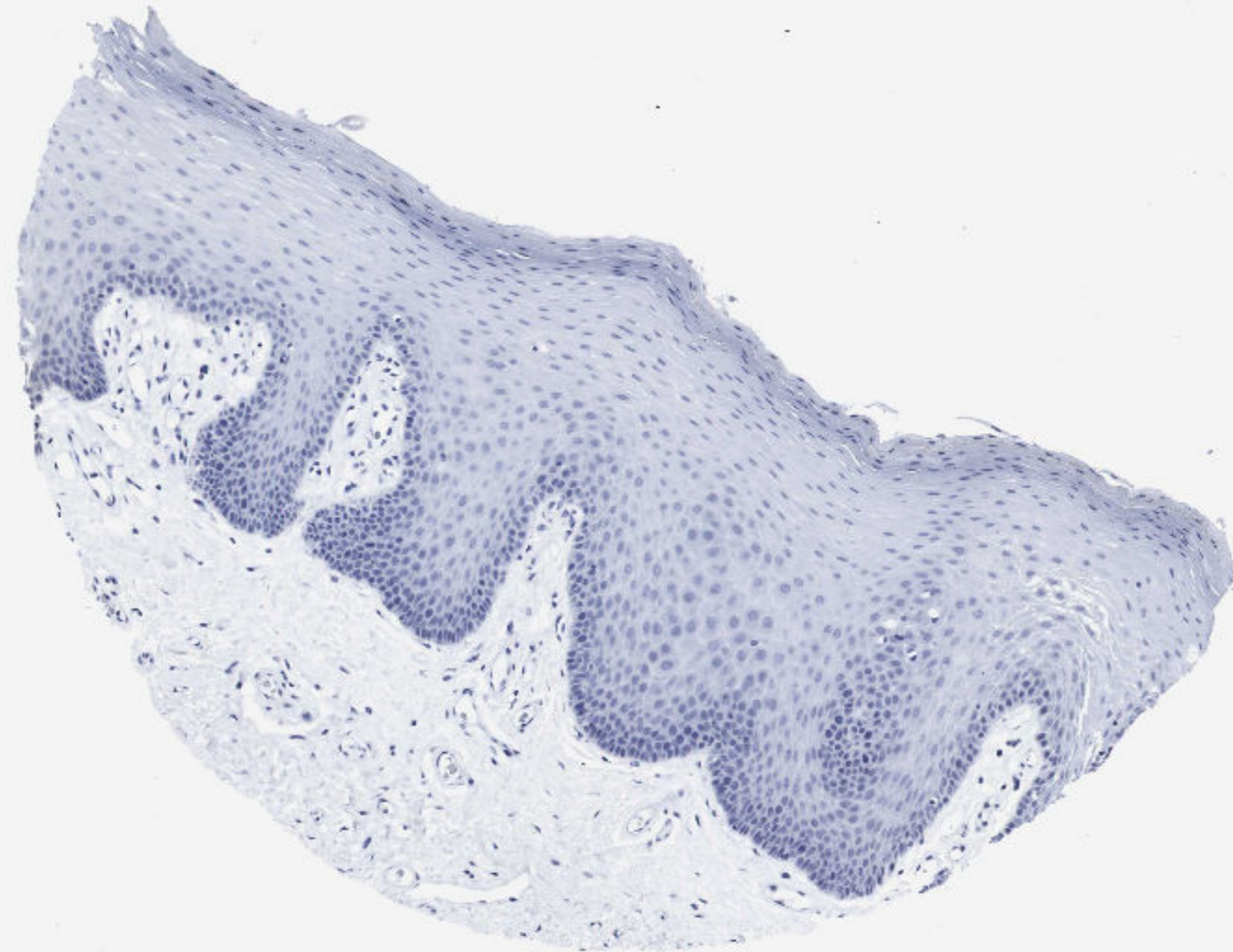

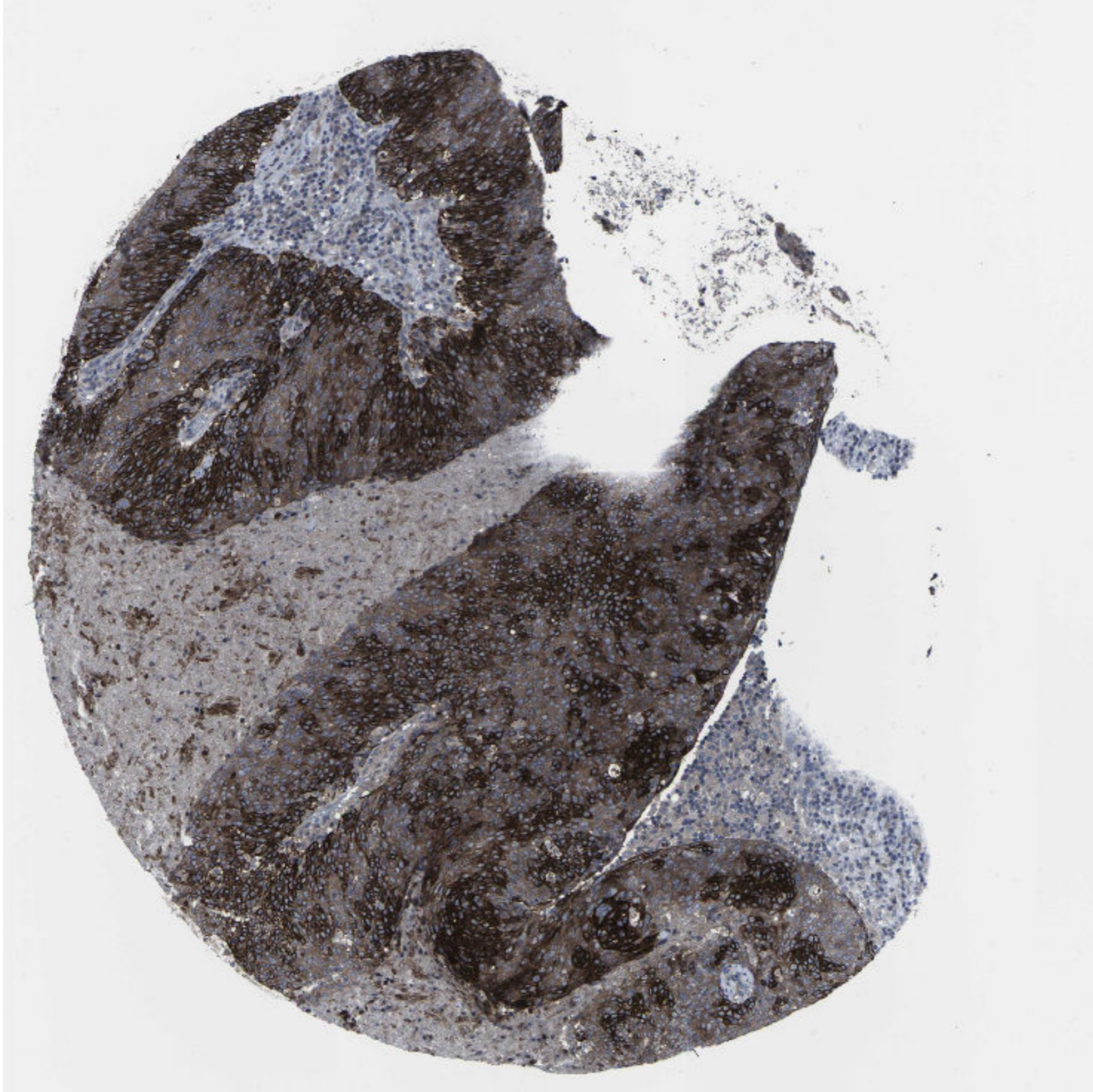

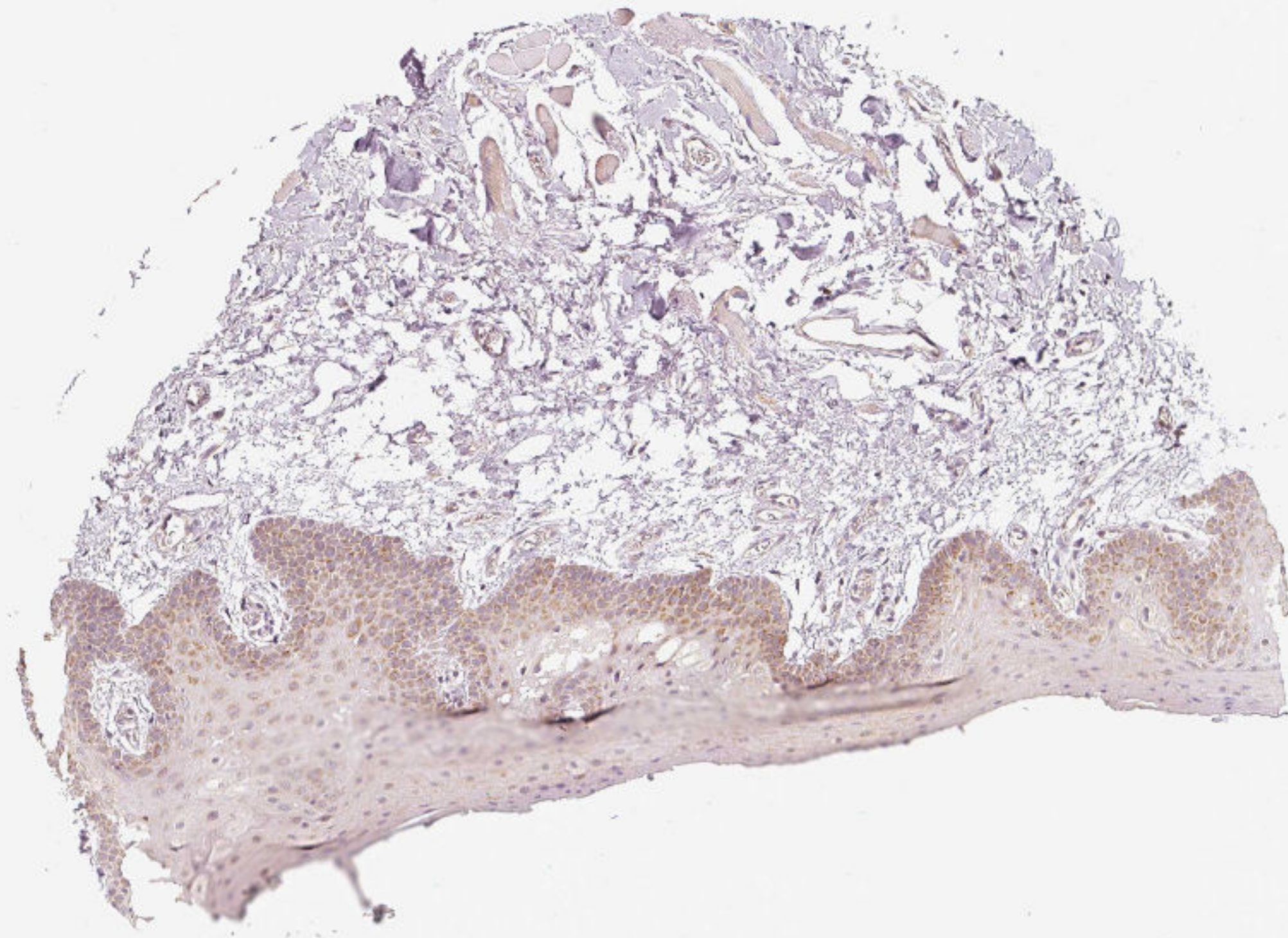

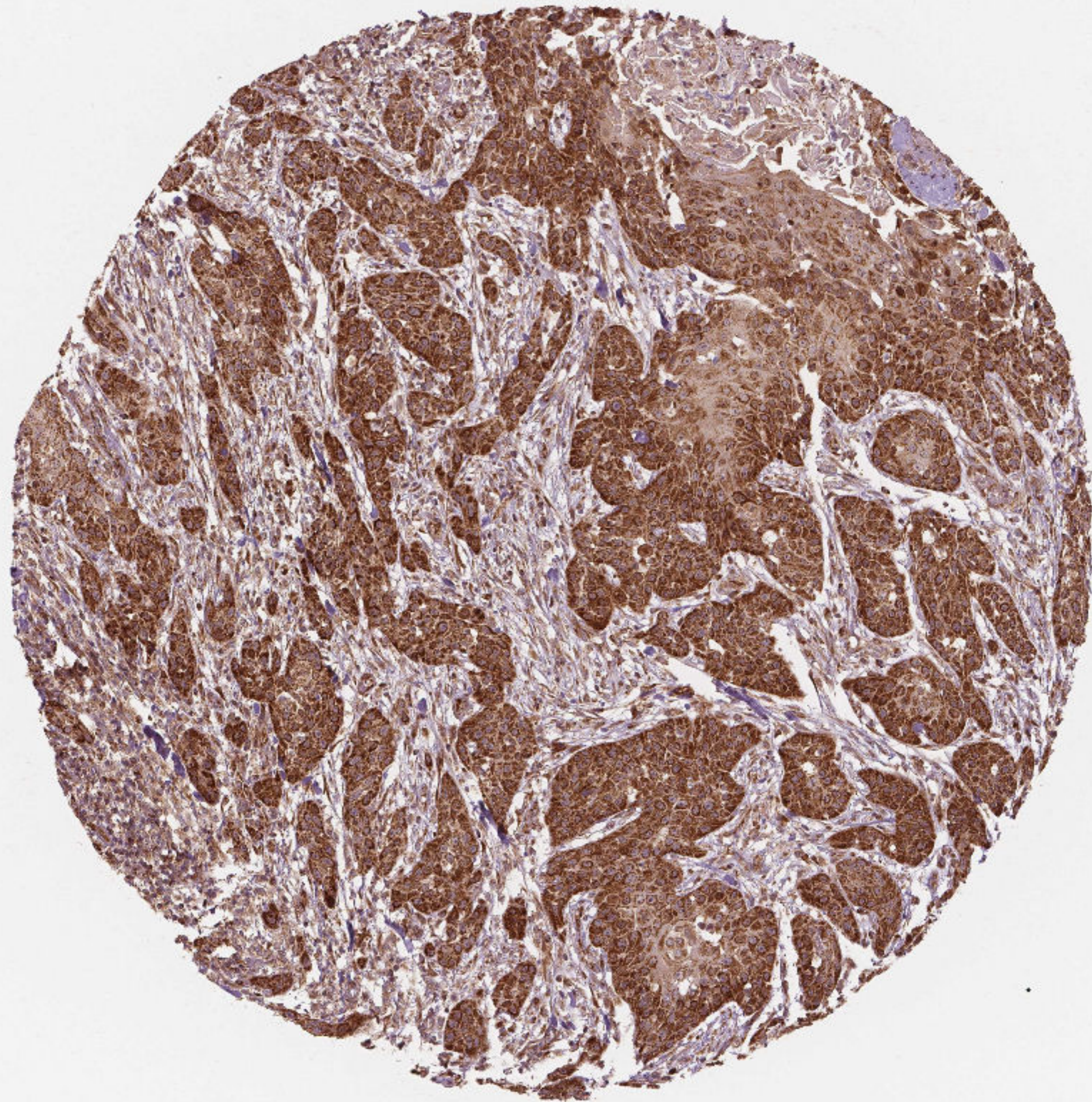

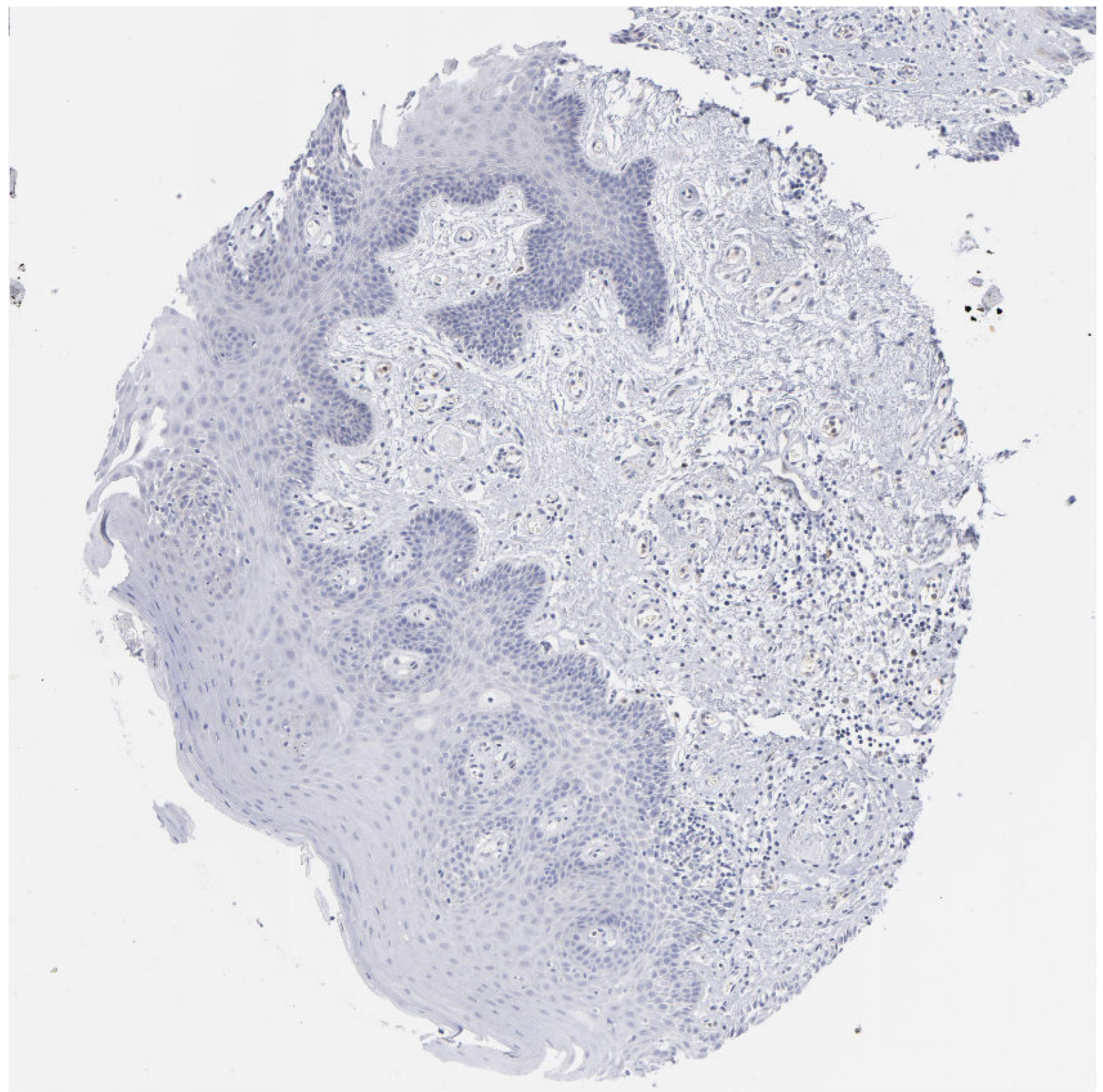

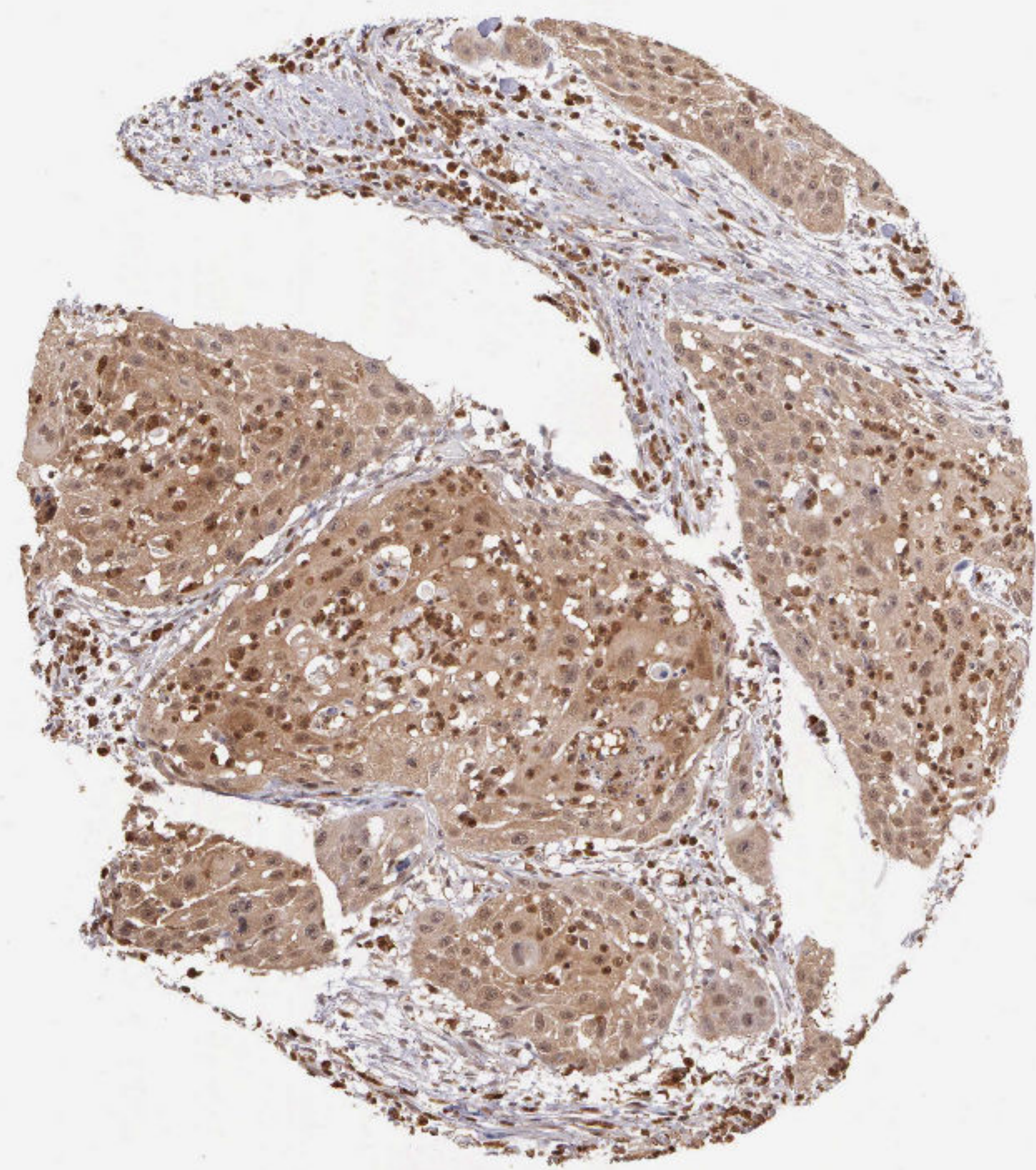

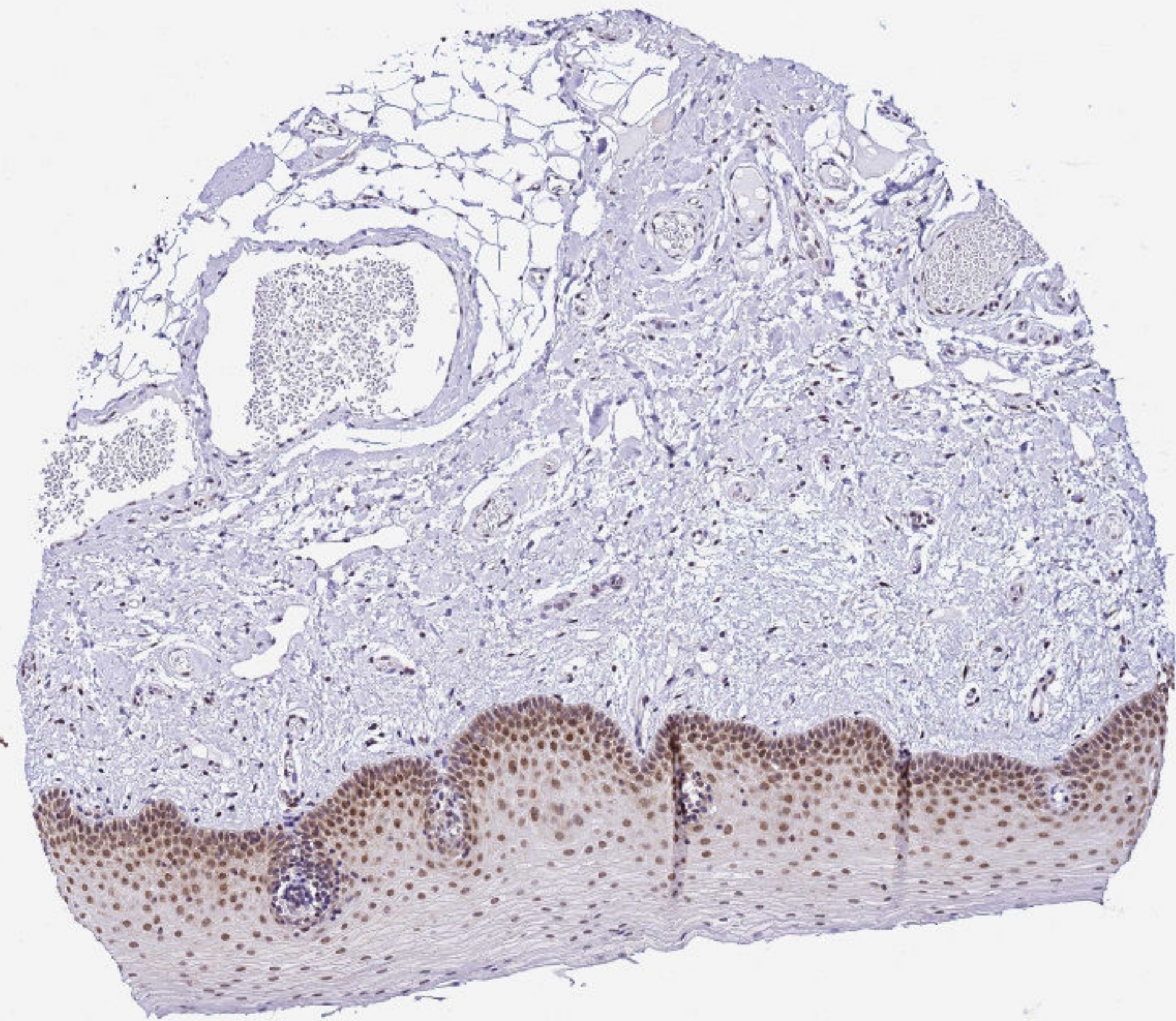

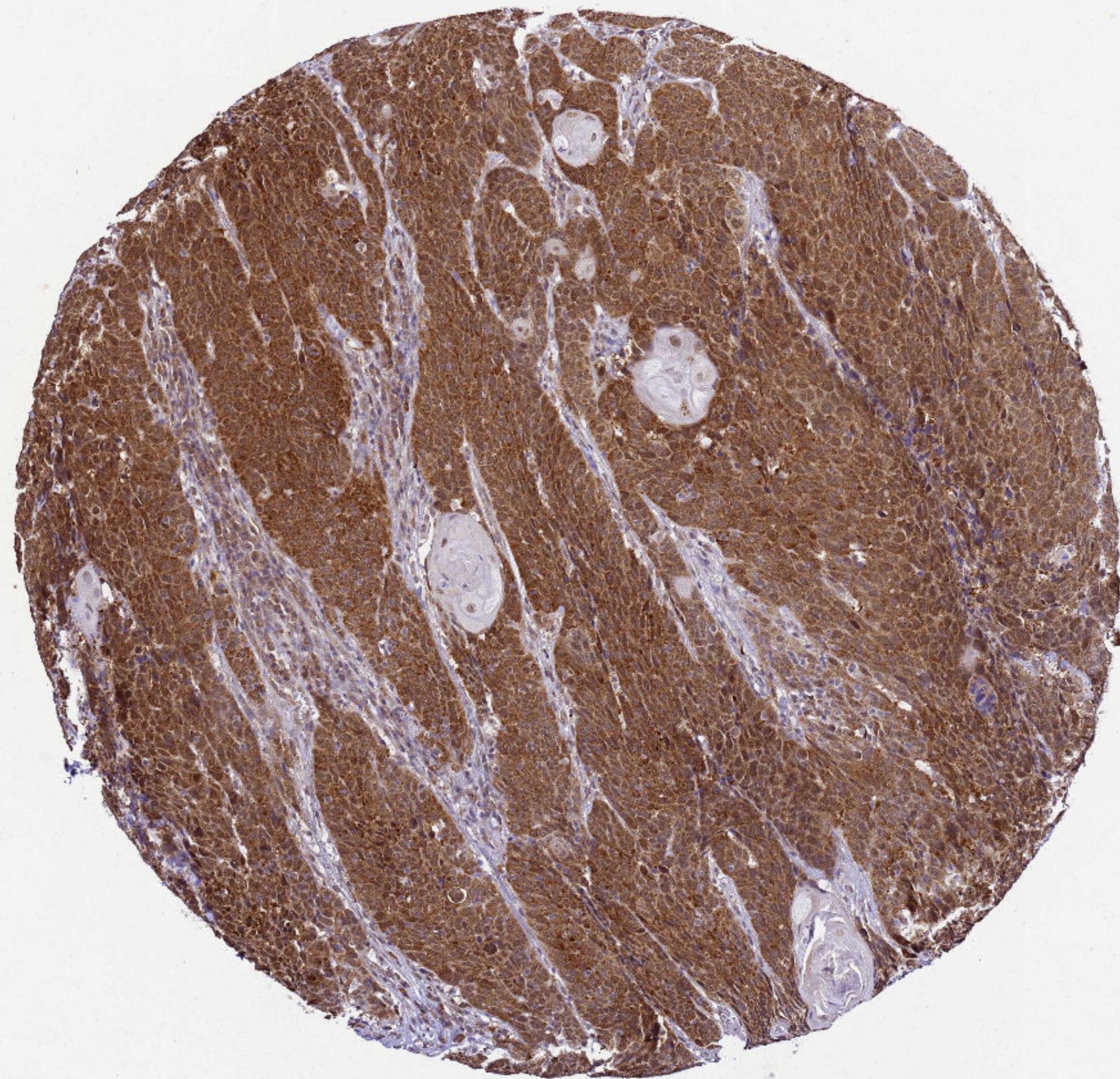

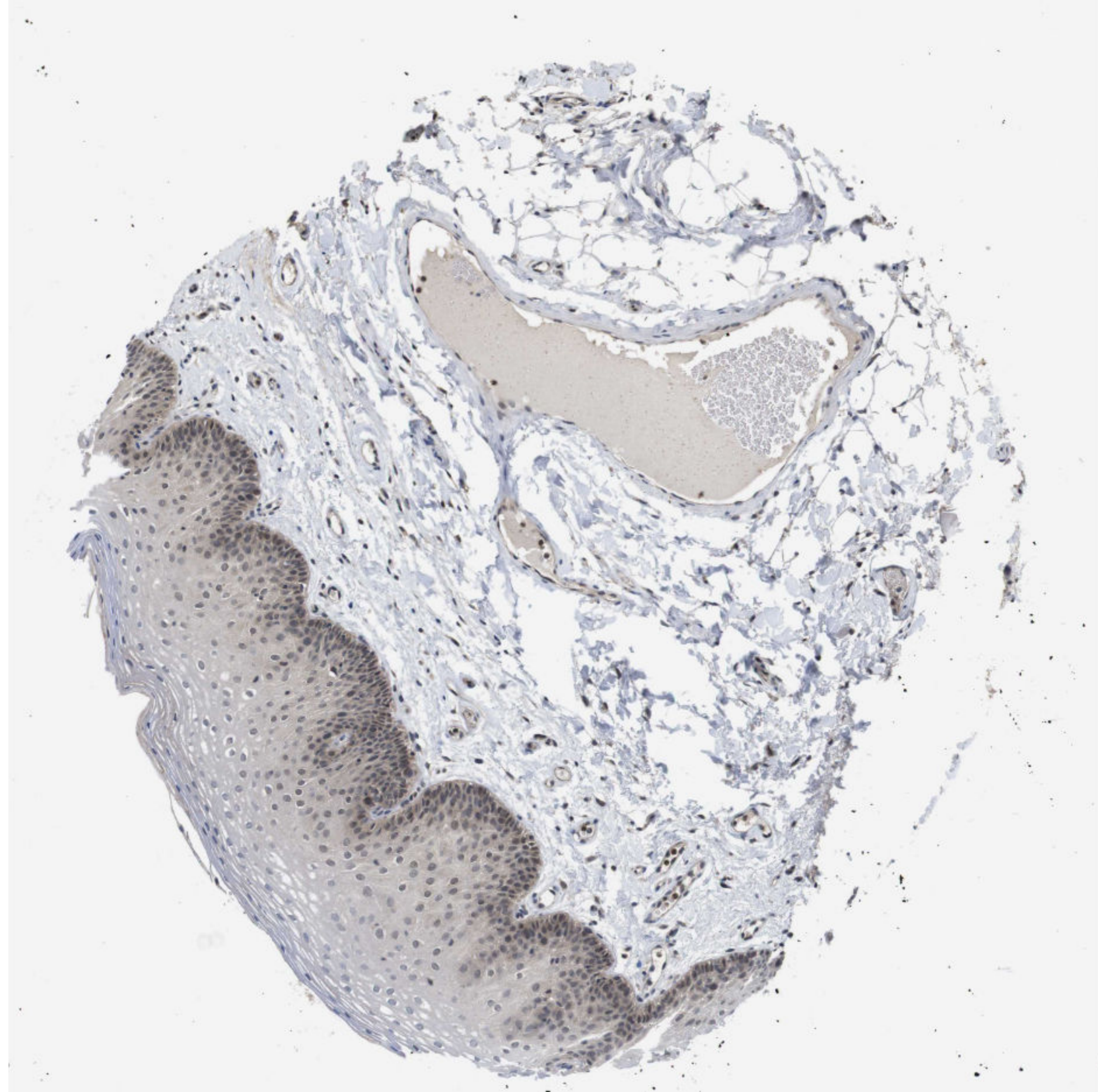

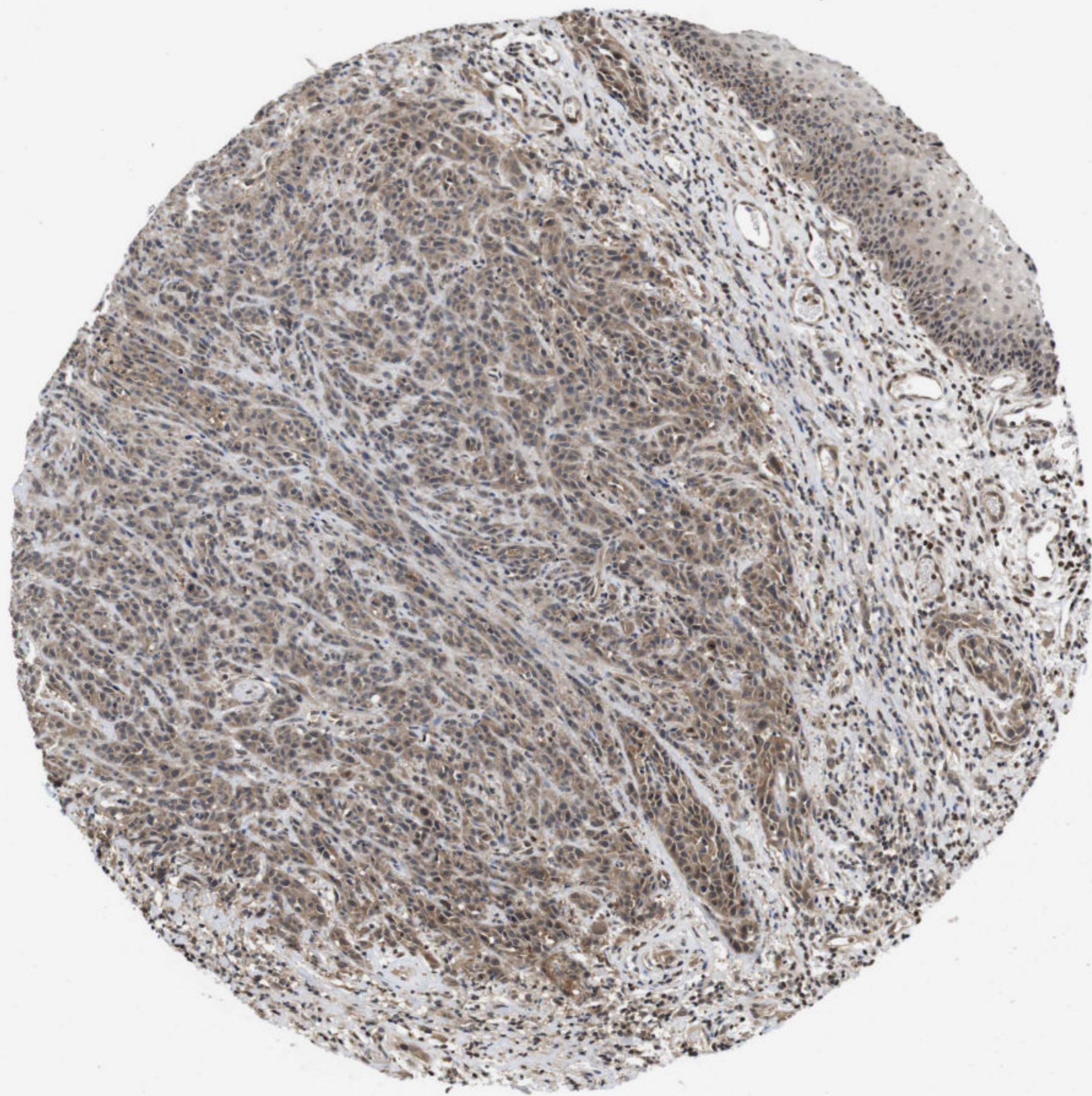

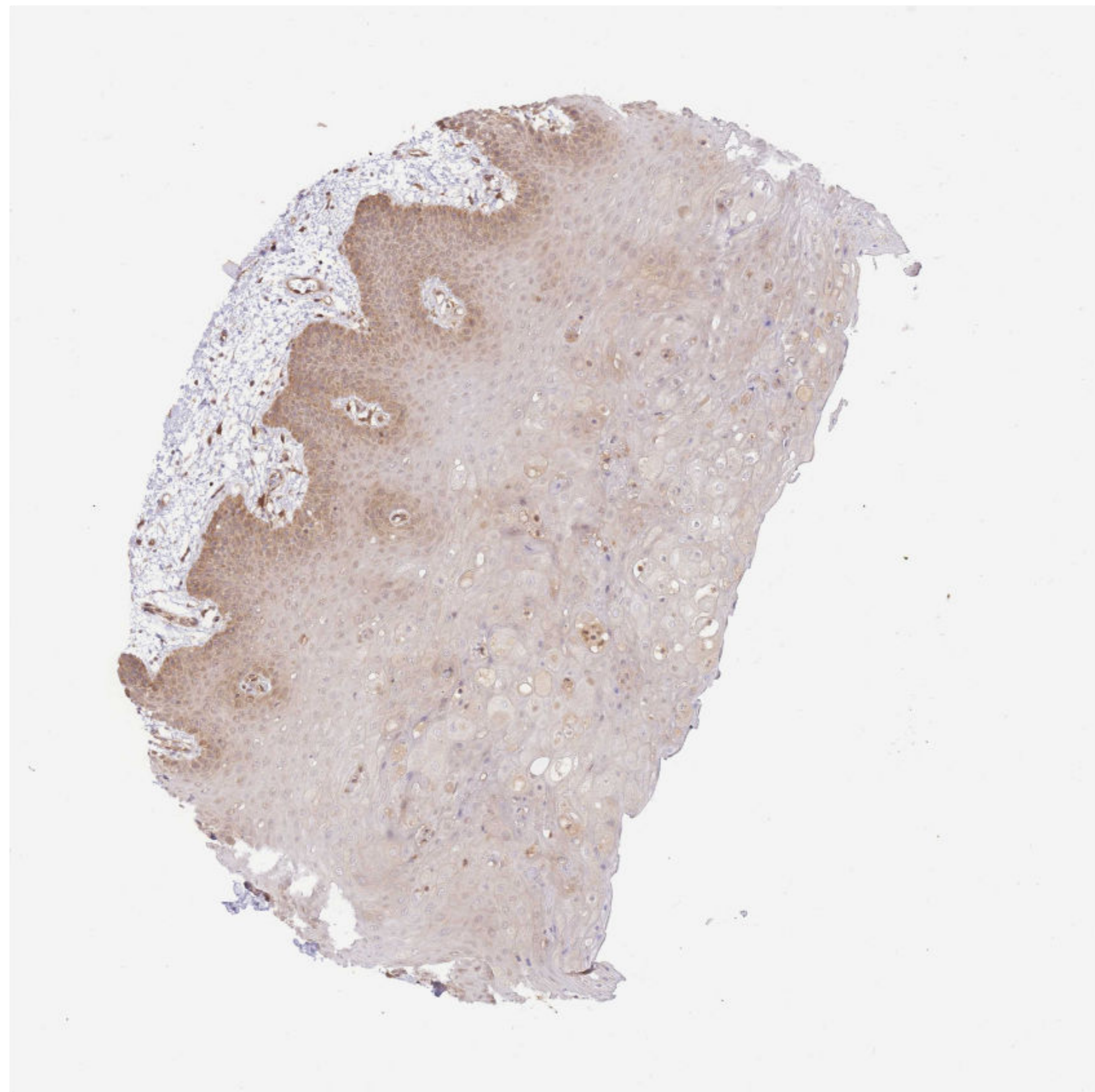

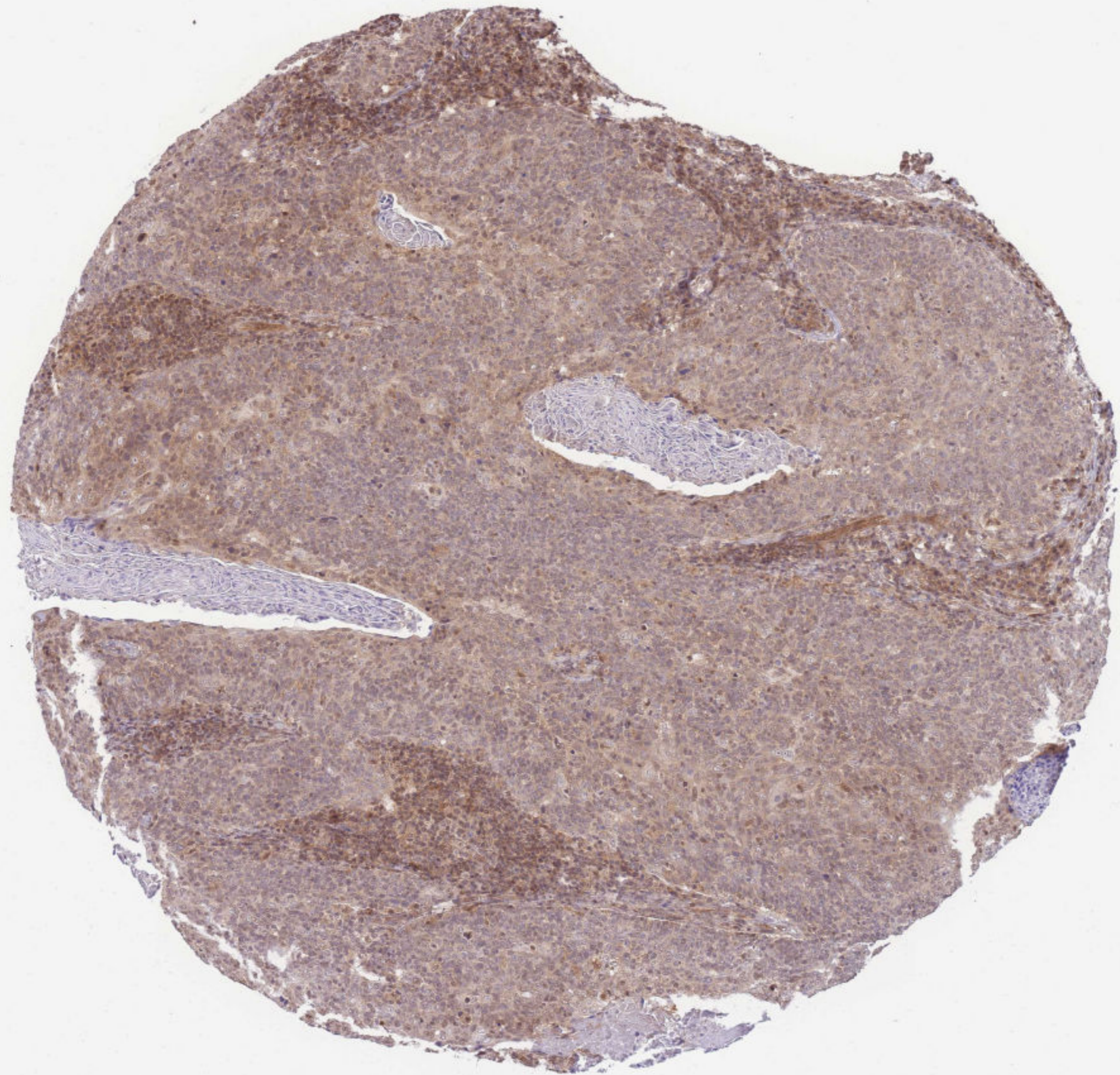

Supplement: Supplementary file 1 [file cancers-15-04539-s001.zip › Figure S1. Higher-resolution original images from the HPA database.pdf]
